# Supplementary material for: IFNγ activates an immune-like regulatory network in the cardiac vascular endothelium
Source: J Mol Cell Cardiol Plus. 2025 Feb 19;11:100289. doi: 10.1016/j.jmccpl.2025.100289 (PMC11919396; doi:10.1016/j.jmccpl.2025.100289)
Supplement: Supplementary file 1 — Supplementary figures [file mmc1.pdf]

SUPPLEMENTAL FIGURES

a

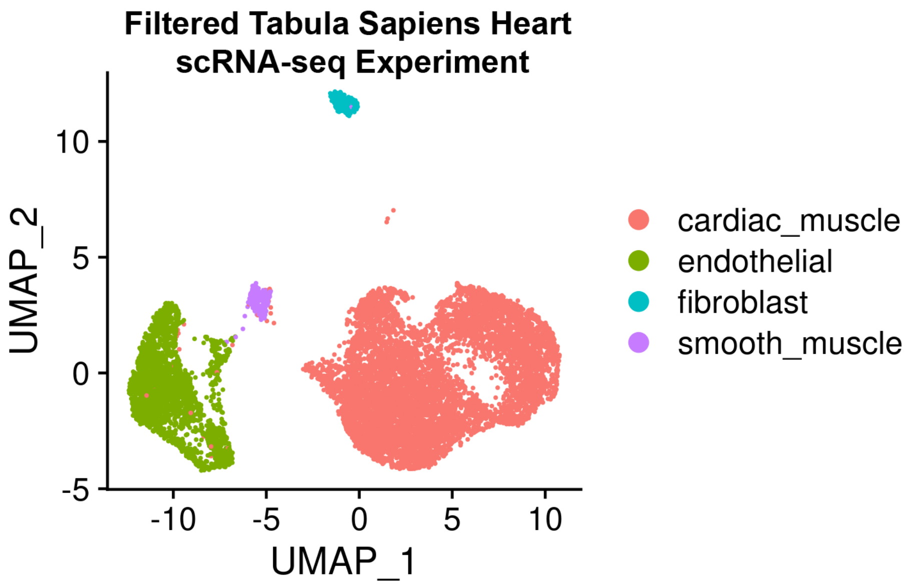

b

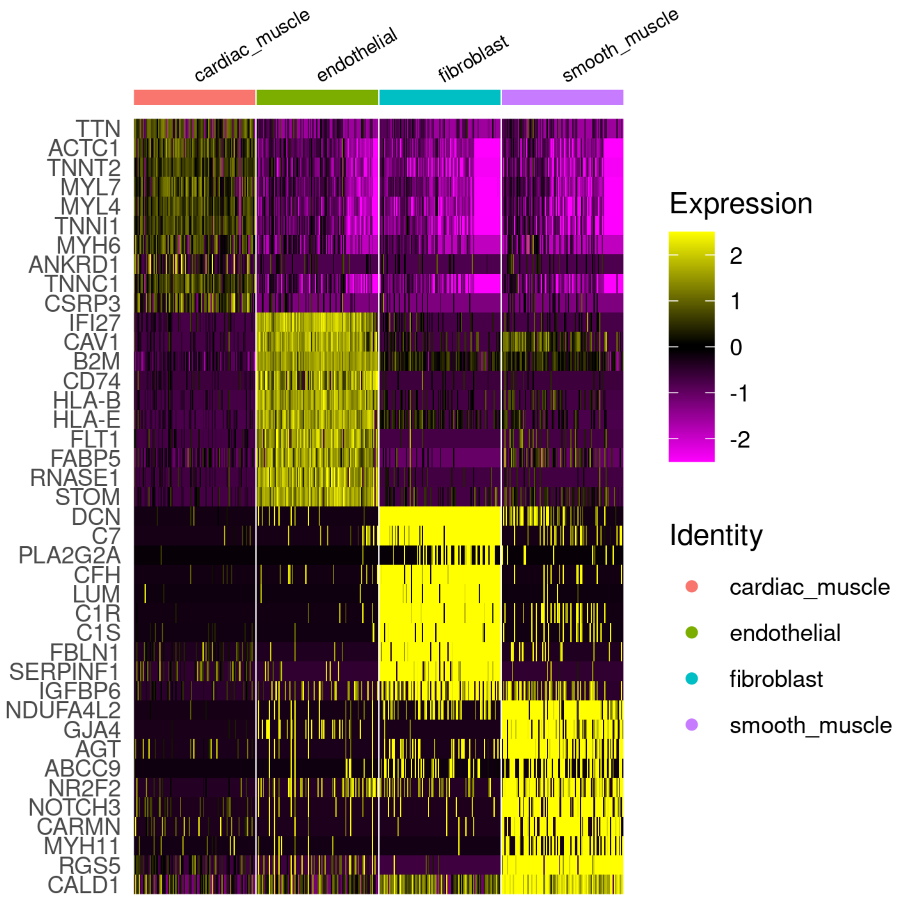

Figure S1. Identification of cardiac cell type markers using the Tabula Sapiens

a) UMAP of the filtered single cell RNA-seq heart dataset from the Tabula Sapiens project. The axes correspond the UMAP coordinates, and each point represents a cardiac cell. The colors correspond to the cell types that are assigned to the cells.

b) Heatmap showing the expression of the top 10 marker genes for the four cell types. The y-axis contains the 40 marker genes, the x-axis are a random subset of 100 cells from each cell type, the cells are filled with the expression of the marker genes.

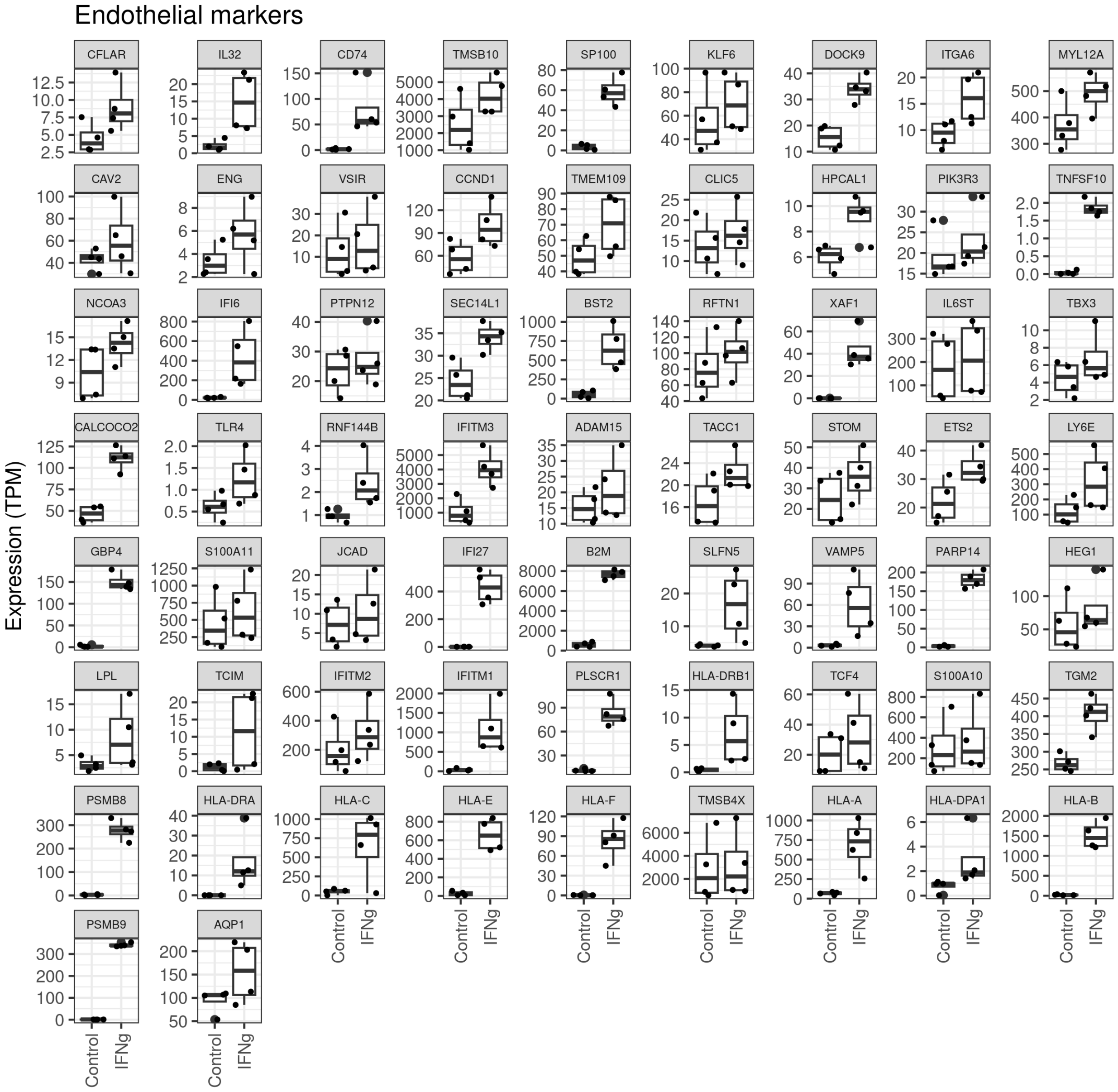

Figure S2. Expression of the IFNγ-upregulated endothelial markers in treated and control CVPCs

Box plots showing the expression of IFNγ-upregulated endothelial markers in treated and control CVPCs. The x-axis is split by the treatment groups ("Control" and "IFNg") and the y-axis is the gene expression (TPM). Each point represents a CVPC RNA-seq sample. The line in the boxes represent the median, and the whiskers represent the 1.5x interquartile range (IQR).

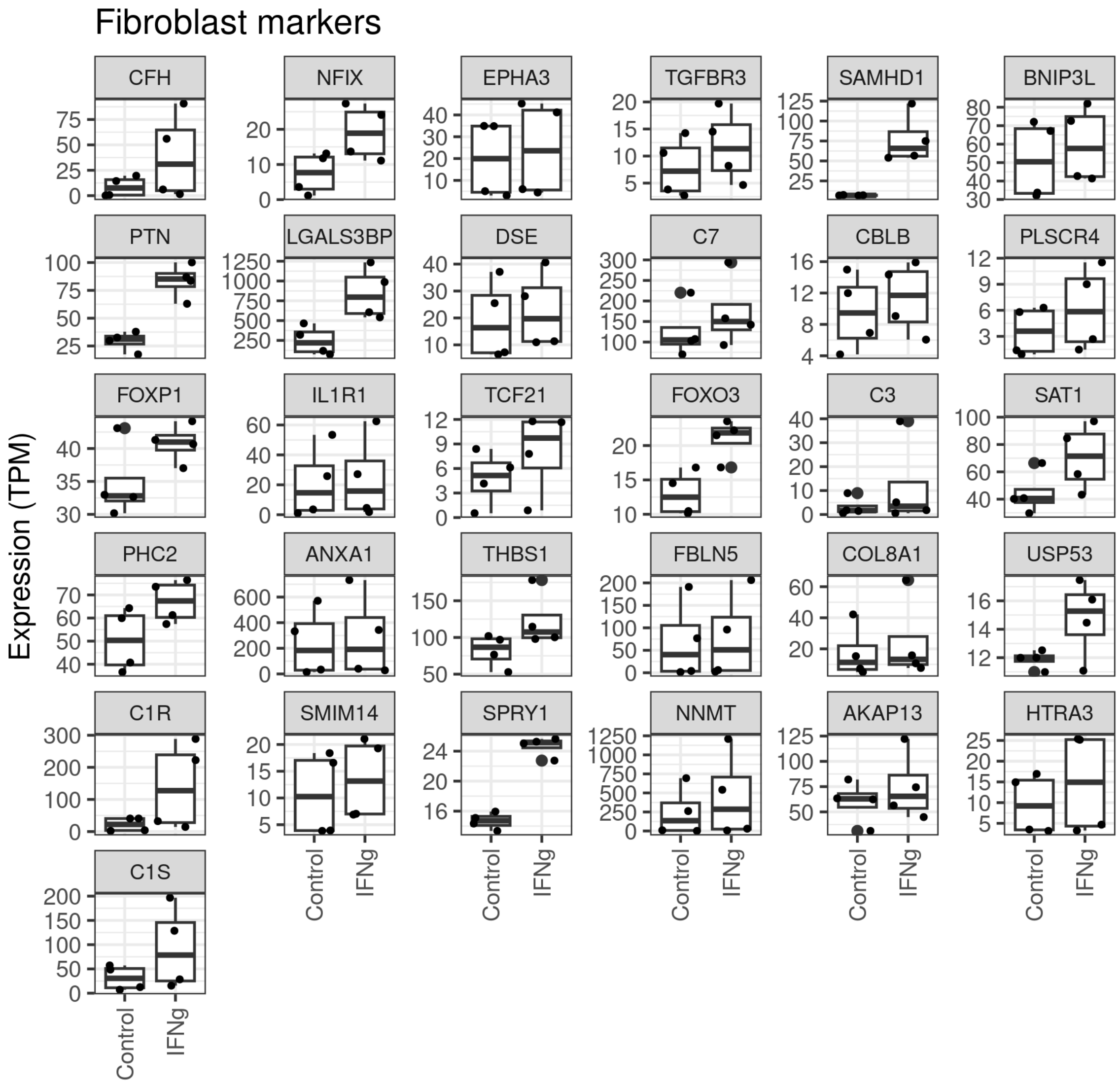

Figure S3. Expression of the IFNγ-upregulated fibroblast markers in treated and control CVPCs

Box plots showing the expression of IFNγ-upregulated fibroblast markers in treated and control CVPCs. The x-axis is split by the treatment groups (“Control” and “IFNg”) and the y-axis is the gene expression (TPM). Each point represents a CVPC RNA-seq sample. The line in the boxes represent the median, and the whiskers represent the 1.5x interquartile range (IQR).
